# Extremely preterm birth and autistic traits in young adulthood: the EPICure study

**DOI:** 10.1186/s13229-021-00414-0

**Published:** 2021-05-06

**Authors:** Helen O’Reilly, Yanyan Ni, Samantha Johnson, Dieter Wolke, Neil Marlow

**Affiliations:** 1 Institute for Women’s Health, University College London, Medical School Building, 74 Huntley Street, London, WC1E 6AU UK; 2 School of Psychology, University College Dublin, Belfield, Dublin 4, Ireland; 3 Department of Health Sciences, University of Leicester, George Davies Centre, University Road, Leicester, LE1 7RH UK; 4 Department of Psychology, and Division of Mental Health and Wellbeing, Warwick Medical School, University of Warwick, Coventry, CV4 7AL UK

**Keywords:** Autistic traits, Broader autism phenotype, Autism spectrum disorder, Preterm birth

## Abstract

**Background:**

A high prevalence of autism spectrum disorder is reported in children born extremely preterm (EP), but an even larger proportion of survivors are affected by subclinical difficulties than meet diagnostic criteria. The aims of this study were to investigate autistic traits associated with the broader autism phenotype in a cohort of young adults born EP, and explore how these traits relate to emotion recognition, empathy and autism symptom presentation in childhood. The prevalence of autism diagnoses was also investigated.

**Methods:**

One hundred and twenty-nine young adults born before 26 weeks of gestation and 65 term-born controls participated in the 19-year follow-up phase of the EPICure studies. In addition to a clinical interview, participants completed the Broader Autism Phenotype Questionnaire (BAPQ), the Empathy Quotient questionnaire, and the Frankfurt Test and Training of Facial Affect Recognition. The Social Communication Questionnaire (SCQ) was completed by the participants’ parents at age 11 years.

**Results:**

EP born young adults scored significantly higher on the BAPQ in comparison with their term-born peers, indicating greater autistic traits. Among EP participants, BAPQ scores were correlated with SCQ scores in childhood (*r* = 0.484, *p* < 0.001). EP young adults had significantly lower scores in emotion recognition and empathy in comparison with controls; however, this effect was mediated by IQ. At 19 years, a diagnosis of autism was reported by 10% of EP participants versus 1.6% of controls, whereas 31% of EP participants scored above the cut-off for the broader autism phenotype in comparison with 8.5% of term-born controls.

**Limitations:**

The high attrition of EP participants from lower socio-economic backgrounds and with lower cognitive functioning may have led to an underrepresentation of those presenting with difficulties associated with autism.

**Conclusions:**

A larger proportion of EP survivors are affected by difficulties associated with autism than have confirmed diagnoses, with a moderate correlation between autism symptom scores in childhood and autistic traits in young adulthood. EP young adults had significantly higher autism symptom scores and a larger proportion had a diagnosis of autism than controls. Screening for autistic traits at set points throughout childhood will help identify those EP individuals at risk of social difficulties who may benefit from intervention.

**Supplementary information:**

The online version contains supplementary material available at 10.1186/s13229-021-00414-0.

## Background

Children born preterm have a higher risk of autism spectrum disorder (hereafter referred to as autism) [1, 2], with risk increasing with decreasing gestational age [3, 4]. The prevalence of autism following birth before 32 weeks gestation is approximately 7–8% [1, 3, 5, 6], while the prevalence in the general population is estimated between 1 and 2% [7]. Biological and environmental factors, such as structural brain changes and negative social experiences [8, 9] due to socio-emotional vulnerability, are proposed to underlie the association between preterm birth and psychopathology, including autism [10, 11].

Johnson et al. [1] examined antecedent risk factors for the diagnosis of autism in the EPICure cohort of extremely preterm (EP; < 26 weeks’ gestation) births. Withdrawn behaviour at 2.5 years of age and cognitive impairment and peer relationship problems at 6 years of age were significant predictors of an autism diagnosis at age 11 years in EP born children; with 8% of this cohort assigned an autism diagnosis. EP born children also had higher autism symptoms scores compared to their term-born peers on a parent screening questionnaire, and 16% of EP children screened positive for autism. Based on this finding, the authors proposed that a greater number of EP children are affected by social and communication difficulties than the small number of EP children that have a diagnosis of autism, but that these difficulties fall outside the diagnostic threshold [1]. This study is in keeping with other research reporting significantly higher autism symptom scores in preterm/low birth weight cohorts versus term-born controls [12–14]. Whether children born EP continue to present with increased symptoms of autism in adulthood or whether those with subclinical symptoms in childhood subsequently meet diagnostic criteria as social complexities increase over adolescence is unknown.

Deficits in social communication and interaction, and restrictive and repetitive behaviours and interests are the core defining features of autism spectrum disorder [15]. The broader autism phenotype (BAP), which is proposed to be a milder presentation of these core features of autism but which fall below the diagnostic threshold, describes the subclinical autistic traits commonly observed in unaffected relatives of autistic individuals [16]. Given the proposal by Johnson et al. [1] that a greater proportion of EP children are affected by subclinical difficulties associated with autism than meet the diagnostic criteria, the BAP profile may be well suited to capturing these subclinical autistic traits observed in EP populations. A study by Eryigit-Madzwamuse et al. [17] reported higher BAP scores in their adult cohort born very preterm (< 32 weeks’ gestation)/very low birth weight (< 1500 g) in comparison with term-born controls. However, to the authors’ knowledge, no prior studies have investigated autistic traits based on BAP features in an EP population.

Two related models of autism, the extreme male brain theory [18] and theory of mind [19], propose that deficits in social interaction and communication, a core defining characteristic of autism, may stem from difficulties in the recognition and understanding of emotions and mental states in others. In line with this, previous studies have reported impairment in emotion recognition [20, 21] and lower self-reported empathy [20, 22] in autistic adults in comparison with controls. Lower empathy [20] and decreased emotion recognition performance [23] are also suggested to be a feature of BAP; however, lower empathy has only been reported in fathers of autistic children [20] and other studies have not reported any difference in emotion recognition between BAP and control participants [20, 21]. Lower empathy competence [24] and increased emotion recognition deficits [25, 26] have also been reported in preterm groups, and these difficulties may be related to the increased autistic traits observed in preterm populations; however, we are unaware of any studies that have examined the association between emotion recognition, empathy and autistic traits in a preterm population.

The aims of the present study were to: (1) investigate if EP young adults present with greater autistic traits compared to term-born controls; (2) determine whether autistic traits in adulthood are correlated with autism symptom scores in childhood; (3) examine between-group differences in emotion recognition and empathy abilities and whether these are related to concurrent autistic symptoms.

## Methods

### Participants

In 1995, all infants born less than 26 weeks’ gestation in the UK and Ireland were invited to participate in the EPICure studies. Follow-up assessments were carried out at 2.5, 6 and 11 years of age [27–29]. Of 306 EP survivors at 19 years of age, 129 (42%) participated in the 19-year follow-up (male = 61; mean age = 19.3). Sixty-five out of 153 (42%) control participants were also followed up at 19 years (male = 25; mean age = 19.2). Control participants were term-born classmates of the EP participants; selection and recruitment are detailed elsewhere [28, 29]. The EP and control participants not assessed at 19 years had either declined participation or did not respond to study invitations. See Table 1 for characteristics of study completers and non-completers.

**Table 1:** Sample characteristics of extremely preterm participants and term-born controls assessed and not assessed at 19 years of age

Variable	EP assessed *N* = 129	EP^a^ not assessed *N* = 177	Difference EP assessed vs. not assessed *p* value^c^	Controls assessed *N* = 65	Controls^b^ not assessed *N* = 88	Difference controls assessed vs. not assessed *p* value^c^	Difference EP vs. controls assessed *p* value^c^
**Characteristics at 19 years**							
Age at 19 year assessment, years Mean (SD)	19.3 (0.6)	–	–	19.2 (0.5)	–	–	0.162
Male Sex *n*/*N* (%)	61/129 (47.3)	87/177 (49.2)	0.747	25/65 (38.5)	39/88 (44.3)	0.440	0.243
Gestational age, weeks							
22 weeks, *n*/*N* (%)	2/129 (1.6)	0/177 (0.0)	0.280	–	–	–	–
23 weeks, *n*/*N* (%)	13/129 (10.1)	13/177 (7.3)		–	–	–	–
24 weeks, *n*/*N* (%)	37/129 (28.7)	60/177 (33.9)		–	–	–	–
25 weeks, *n*/*N* (%)	77/129 (59.7)	104/177 (58.8)		–	–	–	–
Birth weight grams Mean (SD)	740.8 (121.9)	751.4 (108.9)	0.422	–	–	–	–
Parent SES category at 19 years^d^							
Higher professional/managerial *n*/*N* (%)	69/125 (55.2)	–	–	39/64 (60.9)	–	–	0.322
Intermediate occupations *n*/*N* (%)	22/125 (17.6)	–	–	15/64 (23.4)	–	–	
Routine/manual occupations *n*/*N* (%)	22/125 (17.6)	–	–	7/64 (10.9)	–	–	
Other *n*/*N* (%)	12/125 (9.6)	–	–	3/64 (4.7)	–	–	
WASI-II FSIQ^e^ Mean (SD)	85.9 (16.7) (*n* = 127)	–	–	103.9 (10.2) (*n* = 64)	–	–	< 0.001
Cognitive impairment (IQ < 70) *n*/*N* (%)	20/127 (15.8)	–	–	0/64 (0)	–	–	–
**Outcome data at 11 years**							
Parent SES category^d^							
Professional/managerial *n*/*N* (%)	57/110 (51.8)	21/69 (30.4)	0.002	36/60 (60.0)	41/78 (52.6)	0.789	0.657
Intermediate occupations *n*/*N* (%)	27/110 (24.5)	17/69 (24.6)		10/60 (16.7)	13/78 (16.7)		
Routine/manual *n*/*N* (%)	24/110 (21.8)	22/69 (31.9)		13/60 (21.7)	22/78 (28.2)		
Other *n*/*N* (%)	2/110 (1.8)	9/69 (13.0)		1/60 (1.7)	2/78 (2.6)		
KABC MPC^f^ Mean (SD)	86.3 (16.2) (*n* = 121)	80.8 (19.3) (*n* = 95)	0.028	105.7 (11.2) (*n* = 65)	102.9 (10.9) (*n* = 88)	0.111	< 0.001
Cognitive impairment (IQ < 70) *n*/*N* (%)	10/121 (8.3)	19/95 (20.0)	0.012	0/65 (0.0)	0/88 (0.0)	–	0.017
DAWBA Autism Diagnosis^g^ *n*/*N* (%)	10/116 (8.6)	6/84 (7.1)	0.704	0/62 (0.0)	0/81 (0.0)	–	0.017
SCQ total score^h^ Mean (SD)	8.0 (7.8) (n = 109)	7.1 (6.6) (n = 73)	0.414	2.5 (2.5) (n = 58)	3.6 (3.9) (n = 79)	0.061	< 0.001

^a^Denominator: *N* = 306 survivors at 19 years

^b^Denominator: *N* = 153 controls assessed at 11 years

^c^Two-sided p values were calculated using *x* [2] test for categorical variables and t test for continuous variables

^d^SES Socio-economic category classified using UK Office for National Statistics Socio-Economic Classification System: (1) high: higher managerial, administrative and professional occupation; (2) medium: intermediate occupation; (3) low: routine and manual occupation; and (4) other. For participants with missing SES data at 19 years, data collected at 11 years of age were used to minimise data loss

^e^WASI-II FSIQ indicates Full-Scale IQ from the Wechsler Abbreviated Intelligence Scale, 2nd Edition. ^f^KABC MPC indicates Kaufman-Assessment Battery for Children, Mental Processing Composite

^g^DAWBA indicates the Development and Well-Being Assessment

^h^SCQ indicates Social Communication Questionnaire

### Procedure

The psychological assessments at 19 years of age were conducted by the lead author at University College Hospital London as part of a two day medical and psychological investigation. Eleven participants were assessed at home due to personal travel constraints. Participants gave written informed consent to take part. For those participants unable to provide informed consent due to intellectual disability, consent was obtained from a parent or guardian. SES was based on parent occupation [30] and classified as (1) higher managerial, administrative and professional occupation; (2) intermediate occupation; (3) routine and manual occupation; (4) never worked/long-term unemployed (> 1 year), student or unclassifiable due to missing info. The South Central—Hampshire A Research Ethics Committee granted ethical approval for the study (Reference: 13/SC/0514). Although designed as an experimenter blind study, group allocation was inadvertently disclosed during the course of the assessment for the majority of participants.

### Assessment measures at 19 years of age

Participants completed two questionnaires investigating autistic traits: the Broader Autism Phenotype Questionnaire (BAPQ) [16] and the Empathy Quotient (EQ) [22]. The BAPQ was designed to measure the milder subclinical personality and language characteristics associated with autism spectrum disorder seen in the non-autistic relatives of individuals with autism [16]. It produces three subscale scores; aloof personality, rigid personality and pragmatic language, as well as a total score. All scales range from 1 to 6, with higher scores indicating greater autistic traits. Cut-off scores were derived from Sasson et al. [31]; males with a total score ≥ 3.55 and females with a total score ≥ 3.17 were considered to present with subclinical autistic traits (BAP).

The Empathy Quotient (EQ) questionnaire was used to measure empathy. Scores range from 0 to 80 with higher scores indicating greater empathy. Previous research has shown that adults with autism have lower EQ scores compared to control adults, and scores are negatively correlated with autism symptoms [22]. A cut-off score of ≤ 30 was applied in this study to define deficits in empathy seen in 81% of the autism sample investigated [22]. In addition, a clinical interview was carried out as part of the 19-year follow-up assessment; participants and/or their guardian were asked by the examiner if they had ever received a diagnosis of autism or any neurodevelopmental or psychiatric disorder.

The Frankfurt Test and Training of Facial Affect Recognition 2nd Edition (FEFA-2) [32] is a computer based assessment tool of emotion recognition abilities. This emotion recognition task was administered on a laptop computer. A total emotion recognition score and a recognition score for each individual emotion (happy, sad, angry, surprised, disgusted, fearful and neutral) were automatically generated by the programme. Scores range from 0–1, with higher scores indicating greater emotion recognition abilities. Emotion recognition impairment was defined as a total score > 2 standard deviations (SD) below the mean of term-born controls.

General cognitive functioning (IQ) was assessed using the Wechsler Abbreviated Scale of Intelligence 2nd Edition (WASI-II) [33], which estimates a Full Scale IQ score (FSIQ) with a mean of 100 and SD of 15.

### Assessment measures at 11 years of age

Parents completed the Social Communication Questionnaires (SCQ) [34] at the 11-year follow-up, a screening questionnaire for autism. Total scores range from 0 to 39, with higher scores indicating greater symptoms. A cut-off of ≥ 15 is used for identifying significant symptoms of autism. The parents also completed the Development and Well-Being Assessment (DAWBA) [35], a semi-structured diagnostic interview from which participants were assigned a diagnosis of autism.

### Statistical analyses

Stata 15.1 [36] was used for statistical analysis. Mean scores and SDs were calculated for each of the above measures for EP participants and term-born controls. Effect sizes were calculated using Cohen’s *d* with a large effect size classified as ≥ 0.8. Unadjusted and adjusted mean differences between groups and their 95% confidence intervals (CI) were estimated using linear regression models. Lower cognitive functioning and a higher rate of intellectual disability are frequently reported in studies of outcomes following preterm birth [37–39]. Given this consistent finding, adjusted analyses are presented with and without IQ as a covariate to explore the impact of IQ on the outcomes investigated. Odds ratios (ORs) for rates of impairment across all measures for EP participants compared to term-born controls were estimated using binary logistic regression models. The relationships between IQ scores and BAPQ scores were explored using correlation analyses. The relationships between EQ, BAPQ and FEFA-2 scores after adjusting for IQ were analysed using partial correlation analyses. Bonferroni correction was used to adjust for multiple comparisons. McNemar Chi-square test was used to examine any difference in the proportion of EP participants with a diagnosis of autism between the 11-year and the 19-year follow-ups.

Sample size was calculated in G*Power [40] in order to investigate the difference in mean BAPQ scores between EP and control participants. A minimum of 34 participants per group was required, with a power of 90%, a significance level of 5% and a large effect size of 0.8, based on the SCQ group means and SDs from the 11-year follow-up [1]. A post hoc power analysis was also carried out, and a power of 99.9% was reported.

Multiple imputation was applied for the main outcome variable, the BAPQ, to adjust for missing data and selective attrition in the EP group. Missing data were imputed by chained equations using the STATA “MI” procedure. Imputation model variables included both those potentially predicting non-response and/or outcomes in EP populations (sex, birth weight, gestational age in decimal weeks, ethnicity, SES at 11 years and 19 years, neurodevelopmental disability at 11 years and 19 years, IQ scores at 11 years and 19 years, mean SCQ total score at 11 years and BAPQ scores at 19 years). Imputation models were based on the missing at random assumption and twenty imputed datasets. We imputed missing values for EP participants who were assessed at 11 years (*N* = 219).

## Results

### Loss to follow-up

Sample characteristics and loss to follow-up analyses are provided in Table 1. There were no significant differences between EP and control participants at 19 years in age, sex or SES (*p* > 0.05, Table 1). EP participants had significantly lower IQ scores at both 11 and 19 years compared to controls. EP participants assessed at 19 years had significantly higher IQ scores and higher parent SES compared to EP participants not assessed; however, there was no difference between EP young adults assessed and not assessed in the number diagnosed with autism or in mean SCQ total score at 11 years.

### Autistic traits in early adulthood

At the 19-year follow-up, EP participants had significantly higher mean BAPQ total score and BAPQ subscale scores than term-born controls, with medium to large effect sizes (Table 2), indicating greater autistic traits. This difference remained significant after adjustment for age, sex, SES and IQ, but not after Bonferroni correction for multiple comparisons. Multiple imputation was performed as a sensitivity analysis to account for missing BAPQ data for EP participants, see supplemental data Additional file 1: Table S4. 31.2% of EP participants and 8.5% of term-born controls exceeded the BAPQ total score cut-off (adjusted OR 4.87; 95% CI 1.67, 14.15; significant after Bonferroni correction). The percentage of term-born controls that were classified as presenting with BAP characteristics (8.5%) is comparable to the rate found in a community based sample of adults reported by Sasson and colleagues [29]. Based on clinical interview at 19 years, 9.8% (12/123) of EP participants reported ever having a diagnosis of autism compared to 1.6% (1/64) of controls.

**Table 2:** Scores on measures of autistic traits, empathy and emotion recognition abilities in EP young adults and term-born controls

	Extremely preterm			Term controls			EP vs controls						Effect size measured by Cohen’s d
	Male	Female	All	Male	Female	All	Unadjusted		Adjusted for sex, age & SES		Adjusted for sex, age, IQ & SES		
	Mean ± SD	Mean ± SD	Mean ± SD	Mean ± SD	Mean ± SD	Mean ± SD	Mean difference (95% CI)	*p*	Mean difference (95% CI)	*p*	Mean difference (95% CI)	*p*	
BAPQ^a^													
Mean Aloof	2.84 ± 0.86 (n = 53)	2.91 ± 0.85 (n = 64)	2.88 ± 0.85 (n = 117)	2.44 ± 0.78 (n = 22)	2.33 ± 0.79 (n = 39)	2.37 ± 0.78 (n = 61)	0.51 (0.25, 0.77)	< 0.001^+^	0.43 (0.18, 0.68)	0.001^+^	0.32 (0.03, 0.62)	0.033	0.62
Mean Rigid	3.10 ± 0.77 (n = 51)	2.90 ± 0.78 (n = 62)	2.99 ± 0.78 (n = 113)	2.33 ± 0.59 (n = 23)	2.47 ± 0.58 (n = 38)	2.42 ± 0.58 (n = 61)	0.59 (0.33, 0.85)	< 0.001^+^	0.54 (0.28, 0.80)	< 0.001^+^	0.35 (0.05, 0.65)	0.024	0.72
Mean Pragmatic	3.28 ± 0.86 (n = 50)	3.30 ± 0.84 (n = 63)	3.29 ± 0.85 (n = 113)	2.82 ± 0.89 (n = 22)	2.63 ± 0.71 (n = 39)	2.70 ± 0.77 (n = 61)	0.57 (0.35, 0.80)	< 0.001^+^	0.49 (0.27, 0.71)	< 0.001^+^	0.26 (0.01, 0.51)	0.038	0.80
Mean total score	3.07 ± 0.74 (n = 48)	3.03 ± 0.70 (n = 61)	3.05 ± 0.72 (n = 109)	2.50 ± 0.63 (n = 21)	2.49 ± 0.63 (n = 38)	2.49 ± 0.62 (n = 59)	0.55 (0.34, 0.77)	< 0.001^+^	0.47 (0.26, 0.68)	< 0.001^+^	0.28 (0.03, 0.53)	0.026	0.81
EQ^b^													
Mean total score	35.88 ± 11.48 (n = 43)	40.61 ± 13.98 (n = 59)	38.62 ± 13.13 (n = 102)	40.45 ± 13.21 (n = 22)	47.89 ± 10.65 (n = 38)	45.17 ± 12.10 (n = 60)	− 6.55 (− 10.65, − 2.45)	0.002^+^	− 5.55 (− 9.50, − 1.60)	0.006^+^	− 1.03 (− 5.46, 3.39)	0.645	0.51
FEFA2^c^													
Total score	0.72 ± 0.10 (n = 56)	0.76 ± 0.10 (n = 67)	0.74 ± 0.11 (n = 123)	0.78 ± 0.06 (n = 24)	0.84 ± 0.06 (n = 39)	0.82 ± 0.07 (n = 63)	− 0.08 (− 0.10, − 0.05)	< 0.001^+^	− 0.07 (− 0.10, − 0.04)	< 0.001^+^	− 0.01 (− 0.04, 0.02)	0.490	0.80

^a^BAPQ indicates Broader Autism Phenotype Questionnaire

^b^EQ indicates Empathy Quotient

^c^FEFA2 indicates Frankfurt Test and Training of Facial Affect Recognition

*p < 0.05; ^+^Significant after Bonferroni correction (*p* < 0.013 BAPQ; *p* < 0.006 FEFA; no correction for EQ)

### Empathy & emotion recognition outcomes

EP participants scored significantly lower than controls on the EQ and the FEFA-2 total score, indicating greater difficulty in empathy and emotion recognition. These results remained significant after adjustment for sex, age and SES, but not when IQ was added as a covariate (Table 2; see Additional file 2: Table S5 for FEFA-2 recognition scores across individual emotions). EQ score was negatively correlated with BAPQ total score for both groups (EP *r* = − 0.688, *p* < 0.001; Term *r* = − 0.511, *p* < 0.001) and positively correlated with FEFA-2 total score for EP participants only (EP *r* = 0.331, *p* < 0.001; Term *r* = 0.006, *p* = 0.965). BAPQ total score was negatively correlated with FEFA-2 total score for EP participants only (EP *r* = − 0.261, *p* = 0.006; Term *r* = − 0.044, *p* = 0.744). However, when the above analyses were repeated with partial correlations adjusting for IQ, only the correlation between EQ and BAPQ scores remained significant for both groups (see Additional file 3: Table S6). EP participants had significantly higher risk of empathy deficits (EQ score ≤ 30) and impairment in overall emotion recognition compared with controls (Table 3).

**Table 3:** Rates of impairment on measures of autistic traits, empathy and emotion recognition in EP young adults & term-born controls

	Extremely preterm	Term-born controls	EP vs controls Unadjusted		EP vs controls Adjusted for age, sex & SES	
	%(*n*/*N*)	%(*n*/*N*)	OR (95% CI)	*p*	OR (95% CI)	*p*
Empathy Quotient^a^						
Total score						
High empathy	69.6% (71/102)	90.0% (54/60)	–	–	–	–
Low empathy	30.4% (31/102)	10.0% (6/60)	3.93 (1.53, 10.09)	0.004^+^	3.48 (1.32, 9.18)	0.012^+^
BAPQ ^b^						
Mean aloof						
Present	19.7% (23/117)	4.9% (3/61)	4.73 (1.36, 16.46)	0.015	4.72 (1.26, 17.62)	0.021
Mean rigid						
Present	27.4% (31/113)	11.5% (7/61)	2.92 (1.20, 7.10)	0.018	2.53 (1.01, 6.34)	0.047
Mean pragmatic						
Present	39.8% (45/113)	13.1% (8/61)	4.38 (1.91, 10.09)	0.001^+^	4.49 (1.81, 11.12)	0.001^+^
Mean total score						
Present	31.2% (34/109)	8.5% (5/59)	4.90 (1.80, 13.33)	0.002^+^	4.87 (1.67, 14.15)	0.004^+^
FEFA2^c^						
Total						
High recognition	69.9% (86/123)	95.2% (60/63)	–	–	–	–
Low recognition	30.1% (37/123)	4.8% (3/63)	8.60 (2.54, 29.20)	0.001^+^	8.26 (2.39, 28.47)	0.001^+^
Happy						
High recognition	94.3% (116/123)	96.8% (61/63)	–	–	–	–
Low recognition	5.7% (7/123)	3.2% (2/63)	1.84 (0.37, 9.13)	0.455	2.19 (0.42, 11.45)	0.354
Sad						
High recognition	88.6% (109/123)	96.8% (61/63)	–	–	–	–
High recognition	11.4% (14/123)	3.2% (2/63)	3.92 (0.86, 17.81)	0.077	3.77 (0.80, 17.76)	0.093
Fear						
High recognition	90.2% (111/123)	98.4% (62/63)	–	–	–	–
Low recognition	9.8% (12/123)	1.6% (1/63)	6.70 (0.85, 52.78)	0.071	6.80 (0.85, 54.38)	0.071
Angry						
High recognition	79.7% (98/123)	96.8% (61/63)	–	–	–	–
Low recognition	20.3% (25/123)	3.2% (2/63)	7.78 (1.78, 34.02)	0.006	6.67 (1.50, 29.64)	0.013
Surprised						
High recognition	91.1% (112/123)	93.7% (59/63)	–	–	–	–
Low recognition	8.9% (11/123)	6.3% (4/63)	1.45 (0.44, 4.75)	0.541	1.47 (0.43, 4.98)	0.540
Disgusted						
High recognition	76.4% (94/123)	96.8% (61/63)	–	–	–	–
Low recognition	23.6% (29/123)	3.2% (2/63)	9.41 (2.17, 40.87)	0.003^+^	10.52 (2.35, 47.05)	0.002^+^
Neutral						
High recognition	89.4% (110/123)	93.7% (59/63)	–	–	–	–
Low recognition	10.6% (13/123)	6.3% (4/63)	1.74 (0.54, 5.59)	0.350	1.62 (0.48, 5.42)	0.437

^a^Empathy Quotient: (1) high empathy: total score > 30 (reference group); (2) low empathy (total score ≤ 30)

^b^BAPQ cut-off scores from self-reports of Sasson et al. [[31]], reference group for each scale: not present

^c^Frankfurt Test and Training of Facial Affect Recognition 2nd Edition: (1) high recognition (reference group): ≥ -2SD of controls; (2) low recognition: < − 2SD of controls

^+^Significant after Bonferroni correction (*p* < 0.013 BAPQ; *p* < 0.006 FEFA; no correction for EQ)

### Autism symptomology from childhood to adulthood

There was a significant correlation between SCQ total score at 11 years and BAPQ total score at 19 years for EP participants only (EP *N* = 91, *r* = 0.484, *p* < 0.001; Term *N* = 53, *r* = 0.241, *p* < 0.082), indicating a moderate association between autism symptom scores in childhood and autistic traits in early adulthood. Of 110 EP participants with diagnostic data at both 11 and 19 years, 8.2% had a diagnosis of autism at 11 years compared to 10.9% at 19 years (McNemar Chi-square test: ${x}^{2}$=0.317, *p* = 0.508). No term-born controls received a diagnosis of autism at 11 years.

There was a significant negative correlation between FSIQ score on the WASI-II and BAPQ total score among EP participants, with increasing autistic traits associated with decreasing IQ (*r* = − 0.263, *p* = 0.006). Defining cognitive impairment as a FSIQ score less than 70, we found no significant difference in the odds of an autism diagnosis between EP participants with and without cognitive impairment (OR 1.19 (95% CI 0.24, 5.93), *p* = 0.834). Excluding all participants with a cognitive impairment, the odds of an autism diagnosis were not significantly greater in EP participants than controls; however, the confidence interval here is very wide due to the small sample size so this result should be interpreted with caution (OR 6.63 (95% CI 0.83, 53.09), *p* = 0.075).

## Discussion

The results from this longitudinal prospective population-based study found that EP young adults present with significantly greater autistic traits compared to term-born controls. This increase in traits was seen across all BAPQ subscales—aloof personality, rigid personality and pragmatic language, which are proposed to parallel the core deficits of social communication and interaction, and restrictive and repetitive behaviours and interests that define autism spectrum disorder [16]. This finding of greater autistic traits in our EP born young adults is in keeping with a previous study of adults born very preterm/very low birth weight which also used the BAPQ measure [17]. Furthermore, BAPQ total scores at 19 years were positively correlated with SCQ total scores at 11 years in our EP participants, indicating a moderate association between parent-reported autism symptom scores in childhood and self-reported autistic traits in early adulthood in this population. There was no correlation between these scores in term-born controls, which may suggest that the presentation of these autistic traits is more variable in the general population. Alternatively, this may be due to differences in self versus informant reports or simply due to the use of two different measures.

Eight per cent of EP participants were classified as meeting the diagnostic criteria for autism at 11 years [1]. In addition, EP children scored significantly higher on the SCQ compared to their term-born peers, indicating greater symptoms of autism, with 16% reported to have a positive screen for autism on this measure [1]. Based on these findings, Johnson and colleagues [1] proposed that a larger proportion of EP children present with social and communication difficulties than meet the diagnostic criteria for autism. The current study supports this proposal: 31% of the EP young adults scored above the BAPQ cut-off compared to 10% who self-reported a diagnosis of autism. Previous studies have also reported higher autism screening scores in preterm groups in comparison with term-born controls [12–15]. The American Academy of Pediatrics [41] recommends routine screening for autism in all children at 18 and 24 months. However, as screening in EP born children at 2 years of age can result in a high false-positive rate due to comorbid motor disorders or sensory impairments [42, 43], these differential diagnoses should be considered and investigated [44]. Additional screening at key points throughout childhood may help to identify EP children who might be missed on early screening [45], particularly those with intellectual disability or in older children and adolescents who may have been able to mask their early difficulties [44]. Children identified at risk on screening should be followed up with formal diagnostic assessment. Screening for autistic like traits in EP born children will help to identify those with clinical and subthreshold difficulties who could benefit from intervention in social skills training, which are noted to be effective in autistic populations [46, 47]. Furthermore, a screening tool such as the BAPQ, designed to detect milder autistic traits, may be more sensitive at identifying EP born children and adolescents with subthreshold difficulties, in comparison with an autism specific questionnaire measure designed to detect symptoms in the diagnostic range. Despite the high level of autistic traits reported in this EP population, it is important to note that the majority of these EP participants, just less than 70%, did not present with elevated autistic traits.

EP young adults had significantly higher scores across the BAPQ subscales in comparison with their peers. This suggests that EP participants present with symptoms across the autism phenotype rather than difficulties in specific areas of functioning. At the 11-year follow-up, EP children had increased symptoms in social interaction and communication but not in repetitive behaviours compared to term-born controls after adjustment for IQ [1]. The variation in symptom presentation over time may be due to the use of different data sources and measures at each follow-up; for example, the SCQ at 11 years was based on parent report compared to the BAPQ at 19 years based on self-report. The SCQ was developed based on the ADI-R interview and examines autism symptomology in line with ICD-10 and DSM-IV diagnostic criteria [48], thus is a more conservative measure than the BAPQ. Given the BAPQ investigates milder symptoms associated with autism, it may be more sensitive to detect subthreshold repetitive behaviours and restricted interests.

After adjustment for IQ, the correlation between the EQ and BAPQ total scores remained significant for both EP and control participants. This suggests these two questionnaires measure overlapping abilities. The EQ has also been shown to negatively correlate with the Autism Quotient, a measure of autistic traits in adults [22]. The authors suggest that the social domains measured on the Autism Quotient require empathy skills, which explain this relationship. This also suggests how the BAPQ and EQ are connected. The correlation between FEFA-2 total score and total scores on the EQ and BAPQ was mediated by IQ for EP participants.

In the current study, comorbid cognitive impairment was not associated with an increased risk for an autism diagnosis in our EP participants. However at the 11-year follow-up, cognitive impairment at 6 years was a significant predictor of a diagnosis and 69% of EP participants who met the diagnostic criteria for autism had a cognitive impairment [1]. The reason for this discrepancy in findings may be due to the small increase in participants with an autism diagnosis at 19 years; these new cases all had IQ scores above 70, increasing the proportion of participants that fell in the non-impaired range. As outlined in the diagnostic classification of autism [15], symptoms may only clinically manifest when the social demands exceed the capacities of the individual; the higher cognitive capabilities of these participants may have helped them to mask their social difficulties in childhood and only presented later when social demands became more complex over adolescence. Additionally, at 11 years, cognitive impairment was defined using term-born controls as the reference, with scores more than 2 SD below the mean of controls. This analytic strategy was used to account for the Flynn effect associated with using outdated test norms [49]. However, for the 19-year follow-up a contemporary cognitive test was administered (WASI-II) and the traditional clinical cut-off of an IQ score less than 70 was used to define cognitive impairment. This deviation in the cognitive impairment categorisation may account for the variation in findings. Our current findings are in keeping with a recent cohort study of EP born children assessed at 10 years of age, which reported that decreasing gestational age was associated with an increased risk of autism regardless of intellectual ability [50].

Risk factors associated with increased autistic traits or a self-reported diagnosis of autism in our EP young adults were not explored in the current study, as this was previously investigated at the 11-year follow-up, in which male sex, withdrawn behaviour at 2.5 years of age, and cognitive impairment and peer relationship problems at 6 years of age were significant predictors of an autism diagnosis at age 11 years. A recent systematic review reported male sex, being born small for gestational age, and cognitive impairment to be the most consistent risk factors for an autism diagnosis in children born preterm [45]; however, this study was restricted to a narrative synthesis and the authors highlighted that further research is needed to identify risk factors specific to the different preterm gestational age categories. Montagna and Nosarti [11] reviewed a number of studies of very preterm populations which report volume reductions in brain regions involved in social–emotional processing, such as the fusiform gyrus and amygdala, which they propose may underlie social–emotional difficulties in this population. While Ure and colleagues [51] found cystic lesions in cortical white matter on neonatal neuroimaging was associated with a significantly increased odds of a diagnosis of autism in children born very preterm; however, the sample size was small in this study. Further research is needed to determine brain alterations associated with autism in EP populations.

### Limitations

Over 50% of EP and control participants were lost to follow-up. A greater proportion of EP participants assessed at 19 years were from higher SES backgrounds and had significantly higher IQ scores at 11 years in comparison with EP participants not assessed, indicating that those lost to follow-up have likely poorer outcomes. Reassuringly however, the EP participants assessed versus not assessed had similar SCQ total scores at 11 years and the percentage with a diagnosis of autism did not differ significantly. Compared to the original BAPQ findings for EP participants, the multiple imputation results for this measure found EP participants had higher mean scores on the aloof personality and rigid personality subscales as well as on the BAPQ total score, but not on pragmatic language subscale. However, it should be noted that the imputed mean scores were all within ± 1 standard deviation of the original BAPQ total and subscale scores. Nonetheless, loss to follow-up may have led to an underestimation of the groups differences reported.

At 19-year follow-up, the rate of autism in our EP participants and term-born controls was measured based on self-report in clinical interview rather than through the administration of a standardised diagnostic measure. Although this method may be susceptible to response bias, the prevalence of autism in our EP and control participants was comparable between the 11-year follow-up based on the standardised DAWBA parent interview and the 19-year follow-up using self-report. It is also important to highlight that the BAPQ was designed to measure milder autistic traits and does not assess diagnostic features of autism. The lack of a standardised autism diagnostic tool or questionnaire measure is a further limitation of the study.

## Conclusions

EP born young adults presented with increased autistic traits in comparison with their term-born peers, with a significant association to autism symptoms in childhood. A larger proportion of EP survivors are affected by subclinical difficulties associated with autism than meet the diagnostic criteria, and the broader autism phenotype captures this presentation. The higher prevalence of autism in EP born children highlights the need for ongoing screening in this at risk group throughout childhood. An investigation of the effectiveness of social skills training in EP children with clinical and subclinical difficulties, and whether such training reduces concurrent and later symptom presentation, would be beneficial in planning support services for these children.

## Supplementary information

**Additional file 1: Table S4.** Data imputation results for BAPQ outcome variable.

**Additional file 2: Table S5.** FEFA-2 Emotion Recognition Scores and Between Group Differences.

**Additional file 3: Table S6.** Partial correlation between EQ, BAPQ & FEFA, adjusting for IQ.

## Data Availability

Data are available subject to the EPICure Data Sharing Policy (www.epicure.ac.uk) and will be available as part of the RECAP preterm Cohort Platform (https://recap-preterm.eu).

## Abbreviations

Autism: Autism spectrum disorder
EP: Extremely preterm
BAP: Broader autism phenotype
BAPQ: Broader Autism Phenotype Questionnaire
EQ: Empathy Quotient
FEFA-2: Frankfurt test and training of facial affect recognition 2nd edition
SCQ: Social Communication Questionnaire
SES: Socio-economic status
SD: Standard deviation
WASI-II: Wechsler abbreviated scale of intelligence 2nd edition
