# Supplementary material for: Extremely preterm birth and autistic traits in young adulthood: the EPICure study
Source: Mol Autism. 2021 May 6;12:30. doi: 10.1186/s13229-021-00414-0 (PMC8101117; doi:10.1186/s13229-021-00414-0)
Supplement: Supplementary file 1 — Additional file 1: Table S4. Data imputation results for BAPQ outcome variable. [file 13229_2021_414_MOESM1_ESM.docx]

Additional file 1: Table S4. Data imputation results for BAPQ outcome variable

| BAPQ at 19 years | Original (N=109)  Mean (SD) | Imputed (N=219)  Mean (95% CI) |
| --- | --- | --- |
| Mean total score | 3.05 (0.72) | 3.16 (2.99, 3.33) |
| Mean Aloof | 2.88 (0.85) | 2.99 (2.78, 3.20) |
| Mean Rigid | 2.99 (0.78) | 3.40 (3.19, 3.62) |
| Mean Pragmatic | 3.29 (0.85) | 3.08 (2.92, 3.25) |
