# Supplementary material for: Extremely preterm birth and autistic traits in young adulthood: the EPICure study
Source: Mol Autism. 2021 May 6;12:30. doi: 10.1186/s13229-021-00414-0 (PMC8101117; doi:10.1186/s13229-021-00414-0)
Supplement: Supplementary file 2 — Additional file 2: Table S5. FEFA-2 Emotion Recognition Scores and Between Group Differences. [file 13229_2021_414_MOESM2_ESM.docx]

**Additional file 2: Table S5. FEFA-2 Emotion Recognition Scores and Between Group Differences.**

|  | **Extremely Preterm** | | | **Term Controls** | | | **EP females vs males:** | | **EP vs Controls** | | | | | | **Effect size measured by Cohen’s d** |
| --- | --- | --- | --- | --- | --- | --- | --- | --- | --- | --- | --- | --- | --- | --- | --- |
|  | **Male** | **Female** | **All** | **Male** | **Female** | **All** | **Unadjusted** | **Adjusted for age & SES** | **Unadjusted** | | **Adjusted for sex,**  **age & SES** | | **Adjusted for sex,**  **age, IQ & SES** | |  |
|  | **mean±SD** | **mean±SD** | **mean±SD** | **mean±SD** | **mean±SD** | **mean±SD** | **mean difference (95% CI)** | **mean difference (95% CI)** | **mean difference (95% CI)** | ***p*** | **mean difference (95% CI)** | ***p*** | **mean difference (95% CI)** | ***p*** |  |
| **FEFA2^a^**  -Total | 0.72±0.10  (n=56) | 0.76±0.10  (n=67) | 0.74±0.11  (n=123) | 0.78±0.06  (n=24) | 0.84±0.06  (n=39) | 0.82±0.07  (n=63) | 0.05  (0.01, 0.09)  * | 0.05  (0.01, 0.09)  * | -0.08  (-0.10, -0.05) | <0.001^+^ | -0.07  (-0.10, -0.04) | <0.001^+^ | -0.01  (-0.04, 0.02) | 0.490 | 0.80 |
| -Happy | 0.83±0.15  (n=56) | 0.84±0.14  (n=67) | 0.84±0.14  (n=123) | 0.84±0.14  (n=24) | 0.86±0.13  (n=39) | 0.85±0.13  (n=63) | 0.01  (-0.04, 0.07) | 0.01  (-0.04, 0.07) | -0.01  (-0.06, 0.03) | 0.531 | -0.01  (-0.06, 0.03) | 0.509 | 0.01  (-0.04, 0.06) | 0.802 | 0.10 |
| -Sad | 0.67±0.21  (n=56) | 0.71±0.18  (n=67) | 0.69±0.20  (n=123) | 0.74±0.16  (n=24) | 0.76±0.14  (n=39) | 0.75±0.15  (n= 63) | 0.04  (-0.03, 0.11) | 0.03  (-0.04,0.10) | -0.06  (-0.12, -0.00) | 0.034 | -0.05  (-0.11, 0.00) | 0.066 | 0.02  (-0.04, 0.08) | 0.475 | 0.33 |
| -Fear | 0.56±0.27  (n=56) | 0.53±0.30  (n=67) | 0.54±0.29  (n=123) | 0.57±0.24  (n=24) | 0.67±0.25  (n=39) | 0.63±0.25  (n=63) | -0.03  (-0.13, 0.07) | -0.03  (-0.14, 0.08) | -0.09  (-0.18, -0.01) | 0.031 | -0.10  (-0.19, -0.02) | 0.019 | -0.00  (-0.10, 0.09) | 0.945 | 0.34 |
| -Angry | 0.62±0.21  (n=56) | 0.69±0.24  (n=67) | 0.66±0.23  (n=123) | 0.72±0.18  (n=24) | 0.81±0.15  (n=39) | 0.77±0.16  (n=63) | 0.07  (-0.01, 0.15) | 0.06  (-0.03, 0.14) | -0.11  (-0.18, -0.05) | <0.001^+^ | -0.10  (-0.17, -0.04) | 0.002^+^ | -0.03  (-0.10, 0.04) | 0.399 | 0.55 |
| -Surprised | 0.84±0.14  (n=56) | 0.89±0.12  (n=67) | 0.87±0.13  (n=123) | 0.90±0.14  (n=24) | 0.94±0.11  (n=39) | 0.93±0.12  (n=63) | 0.05  (0.00, 0.10)  * | 0.05  (0.00, 0.10)  * | -0.06  (-0.10, -0.02) | 0.004^+^ | -0.06  (-0.10, -0.02) | 0.004^+^ | -0.02  (-0.07, 0.03) | 0.405 | 0.45 |
| -Disgusted | 0.50±0.22  (n=56) | 0.64±0.27  (n=67) | 0.58±0.26  (n=123) | 0.68±0.20  (n=24) | 0.82±0.15  (n=39) | 0.77±0.18  (n=63) | 0.14  (0.05, 0.23)  * | 0.14  (0.04, 0.23)  * | -0.19  (-0.27, -0.12) | <0.001^+^ | -0.19  (-0.26, -0.12) | <0.001^+^ | -0.09  (-0.17, -0.02) | 0.017 | 0.83 |
| -Neutral | 0.89±0.19  (n=56) | 0.95±0.14  (n=67) | 0.92±0.17  (n=123) | 0.95±0.13  (n=24) | 0.95±0.11  (n=39) | 0.95±0.11  (n=63) | 0.05  (-0.01, 0.11) | 0.06  (0.00, 0.12)  * | -0.03  (-0.07, 0.02) | 0.230 | -0.02  (-0.07, 0.02) | 0.318 | 0.05  (-0.00, 0.10) | 0.059 | 0.19 |

. ^a^FEFA2 indicates Frankfurt Test and Training of Facial Affect Recognition.

**p*<0.05; ^+^Significant after Bonferroni correction ( *p* <0.006 FEFA).
