# Supplementary material for: Extremely preterm birth and autistic traits in young adulthood: the EPICure study
Source: Mol Autism. 2021 May 6;12:30. doi: 10.1186/s13229-021-00414-0 (PMC8101117; doi:10.1186/s13229-021-00414-0)
Supplement: Supplementary file 3 — Additional file 3: Table S6. Partial correlation between EQ, BAPQ & FEFA, adjusting for IQ. [file 13229_2021_414_MOESM3_ESM.docx]

Additional file 3: Table S6: Partial correlation between EQ, BAPQ & FEFA, adjusting for IQ

| **EP Participants** |  | | |
| --- | --- | --- | --- |
|  | **n** | **r** | **P** |
| **EQ and BAPQ** | 95 | -0.655 | <0.001 |
| **EQ and FEFA** | 101 | 0.097 | 0.336 |
| **BAPQ and FEFA** | 108 | -0.119 | 0.221 |
| **Term-controls** |  |  |  |
| **EQ and BAPQ** | 56 | -0.511 | <0.001 |
| **EQ and FEFA** | 58  59 | 0.005  -0.043 | 0.971  0.751 |
| **BAPQ and FEFA** |  |  |  |
